# Whole Exome Sequencing Reveals *DYSF*, *FKTN*, and *ISPD* Mutations in Congenital Muscular Dystrophy Without Brain or Eye Involvement

**DOI:** 10.3233/JND-140038

**Published:** 2015

**Authors:** Ozge Ceyhan-Birsoy, Beril Talim, Lindsay C. Swanson, Mert Karakaya, Michelle A. Graff, Alan H. Beggs, Haluk Topaloglu

**Affiliations:** a Division of Genetics and Genomics, The Manton Center for Orphan Disease Research, Boston Children’s Hospital, Harvard Medical School, Boston, MA, USA; b Department of Pediatrics, Pathology Unit, Hacettepe University Children’s Hospital, Ankara, Turkey; c Department of Pediatrics, Neurology Unit, Hacettepe University Children’s Hospital, Ankara, Turkey

**Keywords:** Muscular dystrophies, *DYSF* protein human, Dysferlinopathy, *FKTN* protein human, *ISPD* protein human, neuromuscular diseases, genetic testing

## Abstract

**Background:**

Congenital muscular dystrophies (CMDs) are a genetically and clinically heterogeneous group of neuromuscular disorders. Several genes encoding extracellular matrix, nuclear envelope, sarcolemmal proteins and glycosylation enzymes have been implicated in CMDs. The large overlap of clinical presentations due to mutations in different genes poses a challenge for clinicians in determining disease etiology for each patient.

**Objective:**

We investigated the use of whole exome sequencing (WES) in identifying the genetic cause of disease in 5 CMD patients from 3 families who presented with highly similar clinical features, including early-onset rapidly progressive weakness without brain or eye abnormalities.

**Methods:**

Whole exome sequencing was performed on DNA from affected individuals. Potential functional impacts of mutations were investigated by immunostaining on available muscle biopsies.

**Results:**

Pathogenic mutations in 3 different genes, *DYSF*, *FKTN*, and *ISPD* were identified in each family. Mutation in *DYSF* led to absence of dysferlin protein in patient muscle. Mutations in *ISPD* led to impaired ISDP function, as demonstrated by deficiency of α-dystroglycan glycosylation in patient muscle.

**Conclusions:**

This study highlights the benefit of unbiased genomic approaches in molecular diagnosis of neuromuscular disorders with high clinical heterogeneity, such as the phenotypes observed in our patients. Our results suggest that dysferlin deficiency should be in the differential diagnosis of congenital and rapidly progressive muscular dystrophy, and therefore dysferlin antibody should be in the standard immunohistochemistry panel for muscle biopsies in cases with suspected CMD.

## INTRODUTION

Congenital muscular dystrophies (CMDs) are a group of genetically heterogeneous neuromuscular disorders that present at birth or early infancy with hypotonia and muscle weakness [1]. The broad phenotypic spectrum and large overlap of clinical presentations resulting from different gene mutations pose a challenge for clinicians in making a diagnosis and determining the underlying genetic cause for each patient. Therefore, an unbiased genetic testing approach may be beneficial to determine disease etiology, guide patient clinical care and aid family counseling.

We report 5 affected children from 3 unrelated families who presented with very similar clinical features, including early-onset generalized weakness, severely elevated creatine kinase (CK) levels, and rapid progression of disease. Using whole exome sequencing (WES), we identified mutations in 3 different genes in the 3 families, including novel mutations, in the dysferlin (*DYSF*), fukutin (*FKTN*), and isoprenoid synthase domain-containing protein (*ISPD*) genes. Our results highlight the benefit of WES as a diagnostic tool for neuromuscular diseases with wide clinical spectrum and high genetic heterogeneity, such as the phenotype observed in our patients.

## MATERIALS AND METHODS

### Standard protocol approvals and patient consents

This study was approved by the institutional review board of Boston Children’s Hospital. Written informed consent was obtained from all participants.

### Molecular studies

Whole exome sequencing of DNA from probands and related individuals was performed by the IDDRC Core Next-Gen Sequencing Facility of Boston Children’s Hospital and Harvard Medical School, in collaboration with Axeq Technologies. Samples were enriched for exomic sequences using the Illumina Exome Enrichment protocol and captured libraries were sequenced using Illumina HiSeq 2000 Sequencers. The reads were mapped to the human genome assembly UCSC hg19 using Burrows-Wheeler Alignment (BWA version 0.5.8, http://bio-bwa.sourceforge.net/). Single nucleotide polymorphisms and small insertions/deletions were called with SAMtools (version 0.1.7, http://samtools.sourceforge.net/). The annotated variants were filtered against variations reported on dbSNP132, the 1000 Genomes project November 2010 edition, and the NHLBI Exome Sequencing Project (http://evs.gs.washington.edu/EVS/) databases. Computational tools SIFT [2] and PolyPhen [3] were used to predict the impact of missense, while MaxEntScan [4] and NNSplice [5] were used to predict the impact of splice variants. All reported mutations were confirmed by Sanger sequencing in the patients and carrier parents.

### Immunostaining on muscle biopsies

Muscle histology was examined using standard immunohistochemical staining. Frozen muscle sections from Patient C were fixed in ice-cold acetone for 10 minutes, followed by immunostaining with IIH6 antibody.

## RESULTS

### Case reports

We tested 3 Turkish families with clinical diagnoses of congenital muscular dystrophy by whole exome sequencing.

Two siblings from a consanguineous family (Family A), 14 and 11 years old, presented at 4 months and 6 months of age, respectively with hypotonia and delayed motor milestones. CK levels of the older sibling (a girl) were 728 and 4909 IU/L at 2 and 6 years of age, respectively. She became ambulant at age 2.5 years but lost the ability to walk at 8 years. She developed hip and ankle contractures gradually starting from age 3. Examination at 11 years revealed muscle power of 4/5 for neck flexors, 3+/5 for deltoids, 3+/5 for triceps, 2/5 for quadriceps, and 2/5 for hamstrings. Patients did not have intellectual disability in neurocognitive evaluation. Clinical presentation of the younger affected sibling (a boy) was relatively milder. Despite early onset of hypotonia by 6 months, he continues to walk, albeit with difficulty at age 11. Currently he cannot rise from the floor.

The proband from Family B presented at 8 months of age with hypotonia and delayed motor milestones. CK levels were 2675 IU/L at 1.5 years and 4025 IU/L at 3 years of age. He became ambulant at age 3.5 years and is currently able to walk slowly at 6 years of age. Cranial MRI was normal. Patient did not have intellectual disability in neurocognitive evaluation.

Both affected siblings from Family C presented at 4 months of age with hypotonia and delayed motor milestones. The older sibling is currently nonambulant at 5 years of age. He can barely bear weight on legs. His CK levels were 4581 and 6640 IU/L at age 1.5 and 3.5 years, respectively. Cranial MRI was normal. Patient did not have intellectual disability in neurocognitive evaluation. His younger sibling who is 13 months old now has a maximum ability to sit without support.

Major clinical and muscle biopsy findings in the 5 patients are summarized in Table 1.

### Identification of mutations by WES

We used WES in the affected probands to determine the underlying genetic defects. Samples were sequenced individually. On average, 94% (range 93.3% to 94.3%) of target regions were covered, and 84.4% (range 82.8% to 86.2%) were covered more than 10X. Average median coverage of target regions was 37X (range 30X to 44X across samples). 96.4% of Ref-Seq coding exons were covered. On average, 20,223 (range 19,941 to 20,447) variants were identified in exonic regions. Variants that were absent from or were rare (minor allele frequency <0.1%) in control populations in dbSNP135, 1000 Genomes project (November 2010 edition), and NHLBI Exome Sequencing Project databases, as well as 80 race-matched control chromosomes, were filtered in 82 genes that have been implicated in neuromuscular diseases, including all congenital myopathy, muscular dystrophy, and congenital myasthenia syndrome genes in the literature. We only report variants that were novel or rare in the control populations (<0.1% minor allele frequency that would be consistent with the prevalence of congenital muscular dystrophy) and either 1) were predicted to lead to absent or truncated protein, or 2) have previously been reported in other patients with muscle disease or 3) was in a recessive gene and confirmed to be in trans with a variant meeting one of the above-mentioned criteria. Variants that were rare in the general population but did not meet these criteria were classified as uncertain significance and are not reported.

A homozygous frameshift mutation in the dysferlin gene (*DYSF*): c.2779delG (p.Ala927LeufsX21) that alters the protein sequence beginning from amino acid 927 and is predicted to lead to a premature termination codon 21 amino acids downstream, was identified in the patients from Family A. This variant was confirmed to be heterozygous in the parents. The same variant has previously been reported in ten individuals with hyper-CKemia who were asymptomatic until the second to third decade of life [6], three individuals with LGMD [7, 8], and two siblings with congenital muscular dystrophy who had normal or mildly elevated CK levels at ages 2 and 3 years [9]. The patients in our study had highly elevated CK levels at 2 years old. Arm muscles were preserved in patients described by Paradas et al. [9], however our patients had significant arm weakness. Interestingly, previously reported individuals carrying this variant were of Jews of the Caucasus, Iranian, and Spanish ethnicity, while the patients in our study were Turkish. Loss of function mutations of the *DYSF* gene are an established disease mechanism in limb girdle muscular dystrophy (LGMD) and Miyoshi myopathy, although patients with *DYSF* mutations typically have a later onset and milder disease compared to the patients in our study. Although other patients with earlier onset of muscle weakness have been described, our patients are unique in their presentation with highly elevated CK at an early age and involvement of all proximal muscle groups.

Two compound heterozygous missense variants were identified in the fukutin gene (*FKTN*): c.[915G>C];[920G>A] (p.[Trp305Cys];[Arg307Gln]) in the proband from Family B. The Arg307Gln variant has previously been reported in 2 patients with LGMD and a patient with CMD in compound heterozygous state with frameshift variants in *FKTN* and Trp305Cys variant has been reported in one patient with Muscle-Eye-Brain disease-Fukuyama Congenital Muscular Dystrophy phenotype [10, 11], supporting that they are pathogenic variants. Variants were confirmed to be in trans by parental studies. Based on these reasons, these variants were determined to be pathogenic in this patient.

In both of the two affected siblings from Family C, we identified two novel compound heterozygous mutations in *ISPD*: one missense variant, c.458T>C (p.Ile153Thr) and one variant within the splice consensus sequence that was predicted to lead to altered splicing, c.535-3C>G. Mutations were confirmed to be in trans by parental studies.

No other potentially pathogenic variants were identified in neuromuscular disease-related genes in the patients.

### Confirming the impact of DYSF and ISPD mutations by immunostaining on muscle biopsies

**Fig. 1:**
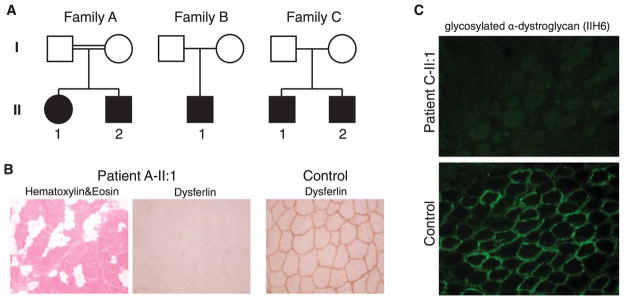
(a) Pedigrees of families A–C. Full black symbol, affected subjects. (b) Dysferlin immunohistochemistry in Patient A-II:1 muscle biopsy and healthy control muscle. (c) Immunostaining with IIH6 antibody for examining glycosylated α-dystroglycan in Patient C-II:1 and control muscle biopsies demonstrating absence of α-dystroglycan glycosylation in the patient muscle.

To confirm the predicted impact of the *DYSF* mutation in Family A, dysferlin immunohistochemistry was performed and it revealed absence of dysferlin protein in the patient muscle (Fig. 1b).

Muscle biopsy was not available from the patient from Family B for α-dystroglycan staining and further studies.

α-Dystroglycan is hypoglycosylated in muscle biopsies from patients with *ISPD* mutations that cause loss of function of the protein [12, 13]. In order to test whether the *ISPD* mutations in Family C caused loss of function of the protein, we analyzed the glycosylation status of α-dystroglycan in the muscle biopsy using IIH6 antibody [12] (Fig. 1c). Staining with IIH6 antibody demonstrated lack of α-dystroglycan glycosylation in patient muscle, suggesting deficiency of ISPD function.

## DISCUSSION

The five patients in our study all presented in the first year of life with rapidly progressing diffuse muscle weakness and highly elevated CK levels at an early age. In Family A, WES revealed a homozygous frameshift mutation in *DYSF*, a gene that is typically associated with a later onset LGMD phenotype and slower disease progression [9, 14, 15]. Reexamining the muscle biopsy in guidance of the genetic test results demonstrated that the mutation led to absence of dysferlin. The results from this case suggest that dysferlin deficiency should be in the differential diagnosis of congenital muscular dystrophy with significantly high CK at an early age. Thus, we suggest that dysferlin antibody should be used as standard in the immunohistochemistry panel for muscle biopsies in cases with suspected CMD.

We identified compound heterozygous mutations in *FKTN* and *ISPD* genes in Families B and C. *FKTN* and *ISPD* are typically associated with CMD or LGMD with variable brain and eye involvement [11, 12, 16, 17]. None of the patients in our study had cognitive or ocular abnormalities. Cranial MRI investigations were normal in all index cases. Patients from Family C with *ISPD* mutations presented with hypotonia and delayed motor milestones at 4 months of age, earlier than previously reported patients with *ISPD* mutations. α-Dystroglycan deficiencies have a diverse clinical spectrum ranging from quite mild with a rather benign LGMD phenotype to severe weakness with profound eye and brain abnormalities. In the vast majority of cases, clinical prediction before genetic analysis may not be accurate. The usefulness of whole exome sequencing for diagnosing various Mendelian diseases have been demonstrated [18]. Our results highlight that unbiased genomic approaches may represent a powerful diagnostic tool for neuromuscular diseases with high clinical, pathological and genetic heterogeneity, such as the phenotype observed in our patients.

## Figures and Tables

**Table 1:** Clinical and histopathologic features of the patients

	Patient	A-II:1	A-II:2	B-II:1	C-II:1	C-II:2
	Age	14 yrs	11 yrs	6 yrs	5 yrs	14 mo
	Sex	Female	Male	Male	Male	Male
	Clinical diagnosis	CMD	CMD	CMD	CMD	CMD
	Histopathologic diagnosis	CMD	NA	CMD	CMD	NA
Pregnancy	Polyhydramnios	No	No	No	No	No
	Fetal movements	Normal	Normal	Normal	Normal	No
Birth	Respiration	Normal	Normal	Normal	Normal	Normal
	Hypotonia	No	No	No	No	No
	Head lag	No	No	No	No	No
	Contractures	No	No	No	No	No
	Fractures	No	No	No	No	No
	High arched palate	Yes	No	NA	NA	NA
Motor milestones	Delayed motor milestones	Yes	Yes	Yes	Yes	Yes
	Sat unsupported	8 mo	9 mo	12 mo	13 mo	13 mo
	Walked unsupported	2.5 yrs	2.5 yrs	3.5 yrs	No	No
Presentation	Age of onset	4 mo	6 mo	8 mo	4 mo	4 mo
	Presenting symptoms	Hypotonia, delayed motor milestones	Hypotonia, delayed motor milestones	Hypotonia, delayed motor milestones	Hypotonia, delayed motor milestones	Hypotonia, delayed motor milestones
	Age of diagnosis	2.5 yrs	2.5 yrs	3 yrs	18 mo	6 mo
Current abilities	Ambulation	Not ambulant since 8 yrs	Walks slowly	Ambulant since 3.5 yrs, walks slowly	Not ambulant	Not ambulant
	Respiration	Normal	Normal	Normal	Normal	Normal
	Feeding	Oral	Oral	Oral	Oral	Oral
Other health issues	Contractures	Hips and ankles (started to develop at 3 yrs)	Ankles (started to develop at 3 yrs)	No	No	No
	Scoliosis	No	No	No	No	No
	Intelligence	Normal	Normal	Normal	Normal	Normal
	Brain MRI	Normal	NA	Normal	Normal	NA
	Cardiac status	Normal	Normal	Normal	Normal	NA
Muscle weakness	Generalized/proximal/distal	Generalized	Generalized	Generalized	Generalized	Generalized
	Facial weakness	No	No	No	No	No
	Ophthalmoplegia	No	No	No	No	No
Serum CK levels (IU/L)	Level 1	728 (2 yrs)	3800 (3 yrs)	2675 (1.5 yrs)	4581 (1.5 yrs)	2016 (9 mo)
	Level 2	4909 (6 yrs)	3747 (4 yrs)	4025 (3 yrs)	6640 (3.5 yrs)	NA
Muscle biopsy findings	Internal nuclei	Yes	No muscle biopsy	NA	Yes	No muscle biopsy
	Fiber size variation	Yes		Yes	Yes	
	Degeneration/regeneration	Yes		NA	Yes	
	Endomysial fibrosis	Yes		Yes	Yes	
	Fatty infiltration	Yes		Yes	Yes	
	IHC (merosin, dystrophin, sarcoglycans (α, β, γ, *δ*))	Positive		Positive	Positive	

**Table 2:** Mutations identified in families A–C

Gene Transcript	Family A	Family B		Family C	
	*DYSF* NM 003494	*FKTN* NM 001079802		*ISPD* NM 001101426	
cDNA	c.2779delG (Hom)	c.915G>C (Het)	c.920G>A (Het)	c.458T>C (Het)	c.535-3C>G (Het)
Protein	p.Ala927LeufsX21	p.Trp305Cys	p.Arg307Gln	p.Ile153Thr	p.(?)
SIFT	NA	Deleterious	Deleterious	Deleterious	NA
PolyPhen	NA	Probably damaging	Probably damaging	Probably damaging	NA
MaxEntScan	Unlikely impact	Unlikely impact	Unlikely impact	Unlikely impact	Likely impact (wt = 10.2, var = 0.7)
NNSplice	Unlikely impact	Unlikely impact	Unlikely impact	Unlikely impact	Likely impact (wt = 0.99, var = 0.5)

## References

[1] Bertini E, D’Amico A, Gualandi F, Petrini S (2011). Congenital muscular dystrophies: A brief review. Semin Pediatr Neurol.

[2] Kumar P, Henikoff S, Ng PC (2009). Predicting the effects of coding non-synonymous variants on protein function using the SIFT algorithm. Nat Protoc.

[3] Adzhubei IA, Schmidt S, Peshkin L, Ramensky VE, Gerasimova A, Bork P (2010). A method and server for predicting damaging missense mutations. Nat Methods.

[4] Yeo G, Burge CB (2004). Maximum entropy modeling of short sequence motifs with applications to RNA splicing signals. J Comput Biol.

[5] Reese MG, Eeckman FH, Kulp D, Haussler D (1997). Improved splice site detection in Genie. J Comput Biol.

[6] Leshinsky-Silver E, Argov Z, Rozenboim L, Cohen S, Tzofi Z, Cohen Y (2007). Dysferlinopathy in the Jews of the Caucasus: A frequent mutation in the dysferlin gene. Neuromuscul Disord.

[7] Nguyen K, Bassez G, Bernard R, Krahn M, Labelle V, Figarella-Branger D (2005). Dysferlin mutations in LGMD2B, Miyoshi myopathy, and atypical dysferlinopathies. Hum Mutat.

[8] Gallardo E, de Luna N, Diaz-Manera J, Rojas-Garcia R, Gonzalez-Quereda L, Flix B (2011). Comparison of dysferlin expression in human skeletal muscle with that in monocytes for the diagnosis of dysferlin myopathy. PLoS One.

[9] Paradas C, Gonzalez-Quereda L, De Luna N, Gallardo E, Garcia-Consuegra I, Gomez H (2009). A new phenotype of dysferlinopathy with congenital onset. Neuromuscul Disord.

[10] Godfrey C, Escolar D, Brockington M, Clement EM, Mein R, Jimenez-Mallebrera C (2006). Fukutin gene mutations in steroid-responsive limb girdle muscular dystrophy. Ann Neurol.

[11] Godfrey C, Clement E, Mein R, Brockington M, Smith J, Talim B (2007). Refining genotype phenotype correlations in muscular dystrophies with defective glycosylation of dystroglycan. Brain.

[12] Willer T, Lee H, Lommel M, Yoshida-Moriguchi T, de Bernabe DB, Venzke D (2012). *ISPD* loss-of-function mutations disrupt dystroglycan O-mannosylation and cause Walker-Warburg syndrome. Nat Genet.

[13] Tasca G, Moro F, Aiello C, Cassandrini D, Fiorillo C, Bertini E (2013). Limb-girdle muscular dystrophy with alpha-dystroglycan deficiency and mutations in the *ISPD* gene. Neurology.

[14] Krahn M, Beroud C, Labelle V, Nguyen K, Bernard R, Bassez G (2009). Analysis of the *DYSF* mutational spectrum in a large cohort of patients. Hum Mutat.

[15] Takahashi T, Aoki M, Tateyama M, Kondo E, Mizuno T, Onodera Y (2003). Dysferlin mutations in Japanese Miyoshi myopathy: Relationship to phenotype. Neurology.

[16] Roscioli T, Kamsteeg EJ, Buysse K, Maystadt I, van Reeuwijk J, van den Elzen C (2012). Mutations in *ISPD* cause Walker-Warburg syndrome and defective glycosylation of alpha-dystroglycan. Nat Genet.

[17] Cirak S, Foley AR, Herrmann R, Willer T, Yau S, Stevens E (2013). *ISPD* gene mutations are a common cause of congenital and limb-girdle muscular dystrophies. Brain.

[18] Yang Y, Muzny DM, Reid JG, Bainbridge MN, Willis A, Ward PA (2013). Clinical whole-exome sequencing for the diagnosis of mendelian disorders. N Engl J Med.

